# Diatomaceous earth as physical control agent for root-knot nematode

**DOI:** 10.1038/s41598-026-64326-w

**Published:** 2026-08-05

**Authors:** Asmahan M. S. Lashein, Marwa A. Sherief, Ezzat M. A. Noweer, Gehan T. El-Bassyouni, Sahar M. Mousa

**Affiliations:** 1 Plant Pathology Department, Nematology Lab, Agricultural and Biological Research Institute, National Research Centre, 33- El-Buhouth St., Dokki, PO Box 12622, Cairo, Egypt; 2 Inorganic Chemistry Department, Advanced Materials, Technology and Mineral Resources Research Institute, National Research Centre, 33 El Bohouth St., Dokki, PO Box 12622, Cairo, Egypt; 3 Refractories, Ceramics and Building Materials Department, Advanced Materials, Technology and Mineral Resources Research Institute, National Research Centre, 33 El Bohouth St., Dokki, PO Box 12622, Cairo, Egypt

**Keywords:** Root-knot nematode, Physical control, Diatomaceous earth, Pest management, Physical damage, Ecology, Ecology, Environmental sciences, Microbiology, Plant sciences

## Abstract

This study evaluated diatomaceous earth (DE), a naturally occurring mineral composed primarily of amorphous silicon dioxide (SiO₂), as a physical control agent against the root-knot nematode *Meloidogyne javanica* infecting eggplant (*Solanum melongena*). Under laboratory conditions, DE was tested against second-stage juveniles (J2) at different concentrations. Among the concentrations of 0.01, 0.05, 0.1, 0.3, 0.5, 1.0, and 2.0 g/L, mortality rates of 100% were observed at concentrations ranging from 0.05 to 2.0 g/L, while 85% mortality was recorded at the lowest concentration (0.01 g/L). In greenhouse conditions, DE was applied at concentrations of 0.05, 0.5, 1.0, and 2.0 g/L using three application methods: root dipping and foliar spray at inoculation time, and soil drench at three distinct time points relative to nematode inoculation. All treatments significantly (*P* ≤ 0.05) reduced nematode infection parameters in a concentration-dependent manner. Root dipping at 2.0 g/L resulted in reductions of 92% (galls), 93% (egg masses), and 92% (adult females). Soil drench applied at the time of inoculation produced reductions of 86%, 85%, and 85% for the same parameters, respectively; while foliar spray resulted in reductions of 73%, 68%, and 73%. Among the application methods, soil drench consistently produced the greatest suppression of J2 populations. In addition to nematode control, DE application was associated with increased plant growth parameters. Soil drench applied one week after inoculation increased root dry weight by 178% at 0.5 g/L and by 185% at 2.0 g/L. These results indicate that DE has potential as a physical control agent against *M. javanica* infecting eggplant and may warrant further investigation for integration into nematode management strategies.

## Introduction

 Eggplant (*Solanum melongena*) is ranked among the top ten fruits and vegetables for phenolic content and antioxidant capacity. Its health-promoting properties, including anticancer activity, cholesterol reduction, and decreased risks of obesity, diabetes, and cardiovascular disease, are largely attributed to high levels of chlorogenic acid^1^. Originating in India, eggplant belongs to the genus *Solanum* and is now cultivated across Asia, Africa, and the Mediterranean, where it can be grown year-round in various seasons^2^. It serves as a critical source of nutrition and income, particularly in developing regions, where it is both a dietary staple and a valuable cash crop for smallholder farmers.

However, eggplant production is threatened by root-knot nematodes (RKNs) of the genus *Meloidogyne*. These microscopic plant-parasitic nematodes infect roots, inducing the formation of galls that impair water and nutrient uptake^3^. The resulting damage leads to stunted growth, yield reductions of 20–50%, and poor-quality harvests. Farmers often respond by increasing water, fertilizer, and chemical inputs, which raises production costs and further undermines profitability^4,5^. *Meloidogyne* spp. are among the most damaging plant-parasitic nematodes worldwide, with over 100 species attacking nearly 3,000 crops and causing billions of dollars in annual agricultural losses^6–9^. In Pakistan, *M. incognita* infests up to 84% of tomato crops in the Pothwar Plateau, with incidence patterns influenced by soil type, cropping systems, and climate a situation mirrored in India, Bangladesh, and Nepal^10^.

Effective nematode management depends on accurate species identification and integrated strategies that combine resistant varieties, cultural practices, biological control, and chemical options^11^. Recent regional studies have explored various approaches; successfully managemet of *M. javanica* in peach was achieved by using neem-based formulations combined with *Pochonia chlamydosporia* and *Purpureocillium lilacinum*^12^. *Bacillus australimaris* LWD 73 combined with moringa leaf extract was reported strong nematicidal activity against M. incognita^13^. Combination of TiO₂ nanoparticles with *Trichoderma polysporum* was suppressed nematodes while enhancing plant physiology and defense^10^. Ecological diversity governs the virulence and distribution of tropical RKN species, with indigenous populations in temperate highlands exhibiting distinct pathogenic patterns threatening tomato production^14^. Despite these advances, the adoption of biological control remains limited due to inconsistent field performance, sustaining reliance on chemical nematicides^15^. Critical gaps persist in field validation, dose optimization, and long-term ecological impact, particularly regarding nano-bio and phyto-based synergies in vegetable crops such as tomato. Historical reliance on chemical nematicides has led to environmental pollution, contaminating soil and water resources and posing risks to human health^16^. In response to these challenges, microbial physical control has emerged as a favorable alternative to chemical interventions. Diatomaceous earth (DE), a natural pesticide derived from fossilized diatoms, works by physically dehydrating insects, offering a solution where chemical pesticides fail due to resistance or toxicity. Effective use requires understanding its limitations its harm to beneficial insects and its ineffectiveness when wet and the critical necessity of using only “Food Grade” DE. With this knowledge, this ancient resource can be a safe and powerful tool for pest management^17–19^. DE functions as a natural, physical control agent against RKNs by employing a mechanical, rather than chemical, mode of action^20^. It is composed of the fossilized remains of diatoms, microscopic algae whose skeletons are made of sharp, glass-like silica. When nematodes or insects crawl over this powder, these microscopic, razor-edged fragments lacerate their waxy outer cuticle, causing the pest to lose moisture and die from dehydration a process completely distinct from chemical poisons that act on the nervous system or internal organs^21^. However, the ongoing discovery and testing of new physical control species is a crucial step forward, paving the way for future solutions that are more effective, affordable, and practical for farmers to use^22^. Beyond direct pest suppression, DE acts as a beneficial soil amendment by improving aeration and water retention, which fosters stronger plant roots and enhances the habitat for nematode-suppressing microbes^23^. DE advantage lies in its safety and multi-functional role as a non-toxic, residue-free material, making DE a key component in integrated strategies for farmers and gardeners moving towards sustainable, ecologically sound pest management^24^. However, despite its documented efficacy against insects, the nematicidal potential of DE against RKNs remains largely unproven, representing a critical knowledge gap.

To date, no empirical evidence exists on whether DE can effectively suppress *Meloidogyne* spp. through physical cuticular disruption, nor on the optimal application methods or concentrations for protecting eggplant.

The objectives of this study were: (i) to evaluate the direct nematicidal effect of DE against *Meloidogyne javanica* second-stage juveniles (J2) in vitro; (ii) to compare the efficacy of root dipping, foliar spray, and soil drench applications at multiple concentrations in reducing nematode infection in eggplant under greenhouse conditions; and (iii) to assess the ancillary effects of DE on plant growth parameters.

Specifically, the following hypotheses were tested: (i) DE induces significant J2 mortality through physical abrasion and cuticular disruption; (ii) root dipping and soil drench applications, particularly at higher concentrations, suppress nematode infection more effectively than foliar spray, as reflected in reduced gall formation, egg mass production, and adult female populations; and (iii) DE application mitigates nematode-induced damage and promotes plant growth, leading to increased root and shoot biomass.

## Materials and methods

### Diatomaceous earth source and characterization

Diatomaceous earth (DE) used in this study was obtained from Al-Fayoum, Egypt, located southwest of Cairo. The raw material was characterized using energy-dispersive X-ray fluorescence (XRF) spectrometry with a Spectro XEPOS 76,004,814 instrument (Boschstraße 10, D-47533 Kleve, Germany). The XRF analysis indicated that silica (SiO₂) was the predominant component, comprising 83.6% of the composition. Additional oxides detected included 4.24% Al₂O₃, 1.07% Fe₂O₃, 6.17% CaO, and trace other oxides totaling 4.86%. Prior to use, the DE was washed with distilled water, dried at 100 °C, and ground to a particle size of > 0.6 mm.

### Collection, identification, and preparation of nematode inoculum

Naturally infected eggplant roots containing *Meloidogyne* spp. were collected from El-Mansouryia village, Giza, Egypt. Roots were washed free of soil, and individual mature females were isolated under a stereomicroscope. Species identification was confirmed by examining perineal patterns from posterior cuticular preparations preserved in 4% formaldehyde, following standard morphological criteria^25^.

After identification, eggplant roots bearing large numbers of egg masses were collected, washed to remove the adhering soil particles, and cut into small pieces, 2–3 cm in size. Then, they are washed with a 10% sodium hypochlorite solution for a few minutes to free the nematode eggs from the surrounding gelatinous masses. They are then poured on a 500-mesh sieve, which trapping the eggs and prevents them from passing through and quickly washed several times with a flowing stream of water to ensure the removal of all traces of hypochlorite solution. Free eggs of identified *M. javanica* were counted and transferred to a 10 cm diameter plastic pots filled with steam sterilized sandy loam soil, which each pot received 3000 eggs and planted to eggplant seedlings. The inoculated pots were placed in a greenhouse and watered as needed. Two months later, infected roots were collected, chopped and used as a source of inoculation for other clean eggplant seedlings. By repeating this procedure, enough quantities of inoculum were obtained and used as the inoculum source for both laboratory assays and greenhouse experiment.

To obtain J2 inoculum, infected roots containing mature females and egg masses were transferred to a plastic washing pan fitted with an aeration system and submerged in tap water. After a three-day hatching period with continuous aeration, the resulting suspension containing freshly hatched J2 was poured through a 325-mesh sieve to retain juveniles which were gently back-washed with a controlled water jet into a 2 L graduated beaker, yielding a concentrated stock suspension. J2 was counted using 1 mL Hawksley counting slide which divided into squares and covered with a glass lid under a light microscope following a zigzag pattern to avoid double-counting. Five replicate 1 ml aliquots were taken for counting, the average number of J2 per milliliter was calculated, and the stock was diluted or concentrated as needed to achieve 200 J2 per 1 mL aliquot for laboratory bioassays and 1000 J2 per 5 mL aliquot for greenhouse pots.

### *In vitro* study

To study the effect of DE on the mortality and recovery of *M. javanica* second stage juveniles, different concentrations of DE were investigated to select the best concentrations that will applied in the further assays.

A) **Bioassay screening**

To evaluate the direct effect of diatomaceous earth (DE) on nematode J_2_, a laboratory bioassay was conducted using nine distinct concentrations: 0.001, 0.005, 0.01, 0.05, 0.1, 0.3, 0.5, 1.0, and 2.0 g/L. The experimental layout and concentration design are summarized in Table 1.

A nematode suspension was prepared containing 200 s-stage juveniles (J2) of the root-knot nematode *Meloidogyne javanica* per milliliter. For each DE concentration, 10 mL of the respective treatment solution was added to 1 mL of the nematode suspension within a 50 mL plastic vial. The control treatment consisted of 10 mL of distilled water combined with 1 mL of the same nematode suspension. All treatments, including the control, were replicated five times to ensure statistical robustness.

Following treatment application, all vials were incubated at 25 ± 1 °C. At three distinct time intervals 24, 48, and 72 h the numbers of viable and dead nematodes in each replicate were counted under a light microscope. Nematode mortality percentages were subsequently calculated for each concentration and time point.

To confirm irreversible mortality, a recovery assessment was performed after the 72-hour exposure period. Surviving nematodes from each treatment were gently transferred to fresh plastic vials containing only distilled water and observed for an additional 24 h. Nematodes that failed to regain normal motility or viability during this recovery period were definitively classified as dead. Final mortality percentages were then adjusted using Abbott’s formula to account for any natural mortality observed in the control group^26^.

**Table 1:** Evaluation of the direct effect of DE concentrations on mortality and recovery % of *Meloidogune javanica* second stage juveniles.

Concentrations of DE (g/Litre)	Percentages mortality			% Recovery	% net mortality
	24 h.	48 h.	72 h.		
**0.001**	52	34	50	69	-
**0.005**	44	43	23	53	-
**0.01**	95	10	100	15	85
**0.05**	100	100	100	-	100
**0.1**	100	100	100	-	100
**0.3**	100	100	100	-	100
**0.5**	100	100	100	-	100
**1.0**	100	100	100	-	100
**2.0**	100	100	100	-	100
**Distilled water (control)**	17	15	10	-	-

Each value represents mean of five replicates:

% mortality was calculated by Abbott’s formula as follow:

$$\:{\%}\:\mathrm{m}\mathrm{o}\mathrm{r}\mathrm{t}\mathrm{a}\mathrm{l}\mathrm{i}\mathrm{t}\mathrm{y}=\:\frac{\mathrm{m}-\mathrm{n}}{100-\mathrm{n}}\mathrm{x}100\:$$

where m = % mortality in the treated sample, and n = % mortality in the control.

$$\:{\%}\:\mathrm{N}\mathrm{e}\mathrm{t}\:\mathrm{m}\mathrm{o}\mathrm{r}\mathrm{t}\mathrm{a}\mathrm{l}\mathrm{i}\mathrm{t}\mathrm{y}\:=\:{\%}\:\mathrm{M}\mathrm{o}\mathrm{r}\mathrm{t}\mathrm{a}\mathrm{l}\mathrm{i}\mathrm{t}\mathrm{y}\:\left(\mathrm{a}\mathrm{t}\:72\:\mathrm{h}\mathrm{r}\mathrm{s}\right)\:-\:{\%}\:\mathrm{R}\mathrm{e}\mathrm{c}\mathrm{o}\mathrm{v}\mathrm{e}\mathrm{r}\mathrm{y}$$

To validate these results, future studies should incorporate a recovery phase to differentiate true mortality from reversible immobilization. Although the reported 100% mortality of *Meloidogyne javanica* juveniles across DE concentrations from 0.05 to 2.0 g/L appears substantial, several methodological concerns warrant consideration.

First, a dose–response relationship spanning two orders of magnitude with no variation in mortality from the lowest tested concentration (0.05 g/L) to the highest (2.0 g/L) is atypical for a physical nematicide. In a valid bioassay, mortality would be expected to increase progressively with concentration. A flat 100% response across such a broad range may indicate insufficient assay resolution or confounding factors, such as desiccation artifacts, osmotic shock, or mechanical damage during handling that could have artificially elevated mortality rates.

Second, the absence of recovery data for treatments achieving 100% mortality represents a notable limitation. Even highly potent physical agents rarely achieve complete population elimination without prolonged exposure or extreme doses. Without a post-exposure recovery period in fresh water, temporary immobilization or quiescence cannot be distinguished from irreversible mortality a distinction that standard nematicidal bioassays typically require.

Third, the contrast between the binary outcome across active concentrations and the modest background mortality of 10–17% in the control group is statistically unusual for biological replicates. This pattern suggests either that the true lethal threshold is ≤ 0.05 g/L, making higher concentrations redundant, or that the experimental design omitted critical intermediate concentrations between 0.01 g/L (95% mortality) and 0.05 g/L (100% mortality).

These findings should therefore be interpreted with caution. Independent replication, a finer concentration gradient, inclusion of a recovery phase, and microscopic verification of cuticular damage are necessary before claims of universal or absolute nematicidal efficacy can be substantiated. Specifically, future studies should incorporate a post-=dexposure recovery period, employ a refined concentration gradient between 0.01 and 0.05 g/L, perform multiple independent replicates with reported standard deviations, and confirm cuticle damage microscopically rather than relying solely on immobility as a proxy for death. Without such validation, the reported 100% mortality across this concentration range should be considered preliminary and subject to potential overestimation.

### Greenhouse studies

A greenhouse experiment was conducted to evaluate the efficacy of selected DE concentrations (0.05, 0.5, 1.0, and 2.0 g/L) against *Meloidogyne javanica* on eggplant. These concentrations had previously induced mortality (100%) of second-stage juveniles (J2) under laboratory bioassay conditions. Three-week-old seedlings of eggplant (*Solanum melongena* L.) cv. Baladi were transplanted into earthen pots (15 cm diameter), each containing 1 kg of solarized sandy loam soil (1:1 w/w). Plants were supplied weekly with a complete N-P-K fertilizer according to standard recommendations for eggplant cultivation. One month after transplanting, the established seedlings were treated with the previous concentrations via five treatment methods, as follow:

1. Root dipping: seedling roots were dipped in 50 mL of each concentration for one hour.
2. Foliar spray: each plant received 50 mL of the respective concentration as a foliar application.
3. Soil drench: each pot received 50 mL of the respective concentration at three time points relative to nematode inoculation: (i) one week before inoculation, (ii) at the time of inoculation, and (iii) one week after inoculation.

Each pot was inoculated with 1000 freshly hatched J2 of *M. javanica*. Non-treated, inoculated plants served as control. All treatments, including the control, were replicated four times. Pots were arranged in a completely randomized design in a greenhouse at temperatures ranging from 28 to 36 °C and relative humidity between 35 and 55%.

Two months after inoculation, the plants were carefully uprooted, and the nematode criteria viz., the numbers of galls, egg masses, adult females and juveniles extracted from the soil according to established protocols^27,28^ were recorded. Also, shoot and root lengths, as well as fresh and dry weights, were measured for each plant. Percentages of reduction in nematode population and increase in plant growth parameters were calculated. The lethal concentration for 50% (LC50) was calculated using Proban software to evaluate the effectiveness of DE concentrations across different application methods against root galls, egg masses, females, and juveniles.

### Statistical analysis

Data were subjected to a one-way analysis of variance (ANOVA). When significant differences were detected, mean separation was performed using Duncan’s Multiple Range Test (DMRT) at a level of 0.05. All statistical analyses were carried out using ASSISTAT software (Version 7.7). The standard error of the mean (SEM) for each treatment was calculated by dividing the sample standard deviation (SD) by the square root of the number or replicates^29^.

### Characterization

The elemental composition of DE was analyzed using an energy-dispersive X-ray fluorescence (XRF) spectrometer (model XEPOS 76004814; Spectro Analytical Instruments, Boschstraße 10, D-47533 Kleve, Germany).

## Results

### Effect of DE concentrations on controlling root knot nematode

**Fig. 1:**
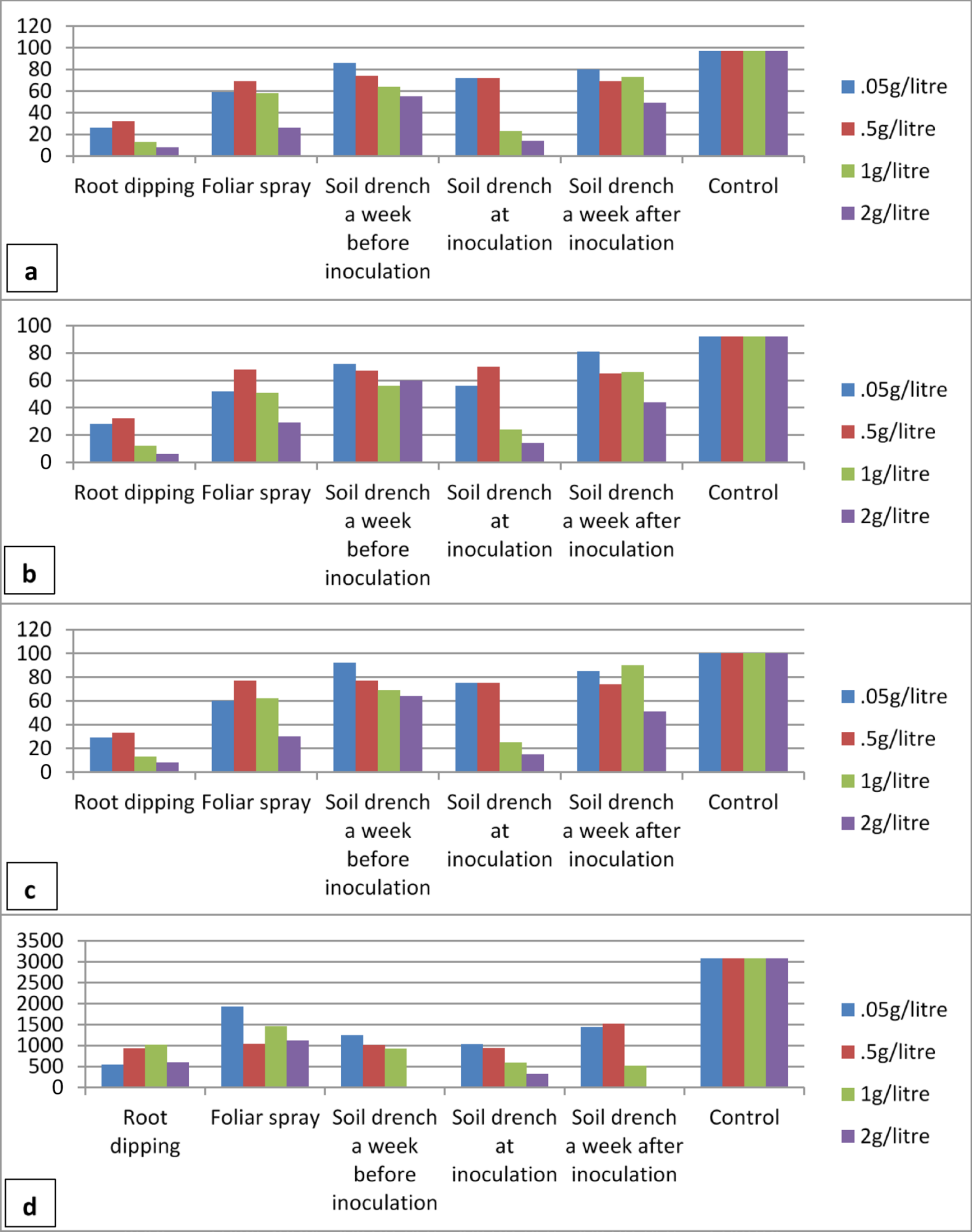
Effect different DE concentrations (0.05,0.5,1.0 and2.0 g/L) on number of galls **(a)**, egg-masses **(b)**, females **(c)** and juveniles **(d)** of root knot nematode, *Meloidogyne javanica* infecting eggplant.

Generally, all DE concentrations and application methods significantly (*P* ≤ 0.05) reduced disease incidence compared to the untreated control, as measured by the number of galls, egg masses, and adult females on eggplant roots, as well as soil juvenile populations in soil (Table 2; Fig. 1). Disease suppression increased with higher DE concentrations, indicating a dose-dependent effect.

Among application methods, root dipping achieved the highest percentages of reduction at the higher concentration of 2.0 g/L, with percentage reductions of 92% (galls), 93% (egg masses), and 92% (females), followed by soil drench at inoculation time which recorded 86% (galls), 85% (egg masses), and 85% (females). Under the same concentration, foliar sprays resulted in reductions of 73%, 68%, and 73% for previous parameters, respectively. As for juveniles, the maximum reduction (100%) was achieved with soil drench applications, one week before and one week after inoculation at 2.0 g/L while drench application at inoculation time produced a 90% reduction.

**Table 2:** Effect of different DE concentrations on controlling root knot nematode, *Meloidogyne javanica* infecting eggplant.

Type of applications	Conc. g/ Liter	No. of galls	% Red.	Nematode population					
				No. of egg masses	% Red.	No. of females	% Red.	No. of juveniles	% Red.
**Root dipping**	0.05	26 cd	73	28 bc	70	29 cd	71	545 de	82
	0.5	32 bc	67	32 bc	65	33 bc	67	930 cd	70
	1.0	13 ef	87	12 cd	87	13 fg	87	1017 cd	67
	2.0	08 f	92	06 d	93	08 g	92	600 de	81
**Foliar spray**	0.05	59 ab	39	52 ab	43	60 ab	40	1928 b	37
	0.5	69 ab	29	68 ab	26	77 ab	23	1040 cd	66
	1.0	58 ab	40	51 ab	45	62 ab	38	1458 bc	53
	2.0	26 cd	73	29 bc	68	27 cd	73	1117 cd	64
**Soil drench a week before inoculation**	0.05	86 a	11	72 ab	22	92 a	8	1245 bc	60
	0.5	75 ab	23	67 ab	27	77 ab	23	1015 cd	67
	1.0	64 ab	34	56 ab	39	69 ab	31	923 cd	70
	2.0	55 ab	43	60 ab	35	64 ab	36	0 f	100
**Soil drench at inoculation**	0.05	72 ab	26	56 ab	39	75 ab	25	1031 cd	66
	0.5	72 ab	26	70 ab	24	75 ab	25	937 cd	70
	1.0	23 de	76	24 bc	74	25 de	75	593 de	81
	2.0	14 ef	86	14 cd	85	15 ef	85	323 ef	90
**Soil drench a week after inoculation**	0.05	80 ab	18	81 a	12	85 ab	15	1438 bc	53
	0.5	69 ab	29	65 ab	29	74 ab	26	1518 bc	51
	1.0	73 ab	25	66 ab	28	90 a	10	515 de	83
	2.0	49 ab	49	44 ab	52	51 ab	49	0 f	100
**Control**	-	97 a	-	92 a	-	100 a	-	3077 a	-
**F value**	-	3.2060	-	2.8421	-	3.4996	-	8.6127	-
**P value**	-	0.00069	-	0.00790	-	0.00148	-	0.00032	-

Data represent the mean of four independent replicates. Statistical comparisons were performed using Duncan’s Multiple Range Test at the 5% significance level, where means sharing the same letter(s) are not significantly different.

Abbreviations: Conc., Concentration; Red., Reduction.

### Evaluating the relative toxicity of application methods of DE against root knot nematode infecting eggplant

The lethal concentrations of 50% (LC_50_) of the root knot nematode, *Meloidogyne javanica* population was determined to evaluate the effectiveness of diatom concentrations using different application methods against root galls, egg masses, females as well as nematode juveniles (Table 3). The obtained results showed that, toxicity of DE concentrations when applied as a root dipping and soil drench at inoculation was more efficacy than the other application methods, which the LC_50_ was (0.94, 0.38 and 0.43) and (0.79, 0.83 and 0.70), respectively against root galls, egg masses, females, followed by foliar spray (1.2, 1.1 and 1.2), and decreases in other methods of addition. While it was more efficacy against juveniles (0.51, 0.50 and 0.55) when applied as soil drench before and after a week of inoculation, and root dipping respectively.

**Table 3:** The toxicity of DE concentrations by different applying methods against the root knot nematode, *Meloidogyne javanica* criteria.

Type of applications		Conc. (g/Litre)	%Reduction in galls	%Reduction in egg masses	%Reduction in females	%Reduction in juveniles
**Root dipping**		0.05	73	70	71	82
		0.5	67	65	67	70
		1.0	87	87	87	67
		2.0	92	93	92	81
**LC**_**50**_		-	0.94 (0.23–0.53)	0.38 (0.23–0.49)	0.43 (0.29–0.54)	0.55 (0.31–0.72)
**Slop ± SE**		-	1.8 ± 0.3	2.0 ± 0.4	2.2 ± 0.4	1.4 ± 0.3
**Foliar spray**		0.05	39	43	40	37
		0.5	29	26	23	66
		1.0	40	45	38	53
		2.0	73	68	73	64
**LC**_**50**_		-	1.2 (1.0–1.5)	1.1 (0.94–1.4)	1.2 (1.0–1.4)	1.2 (0.99–1.7)
**Slop ± SE**		-	2.3 ± 0.3	1.8 ± 0.3	2.3 ± 0.3	1.4 ± 0.3
**Soil drench**	**A week before inoculation**	0.05	11	22	8	60
		0.5	23	27	23	67
		1.0	34	39	31	70
		2.0	43	35	36	100
	**LC**_**50**_	-	2.9 (1.9–11.4)	3.0 (2.0–9.0)	2.5 (1.7–2.7)	0.51 (0.37–0.63)
	**Slop ± SE**	-	1.1 ± 0.3	1.3 ± 0.3	1.3 ± 0.3	2.1 ± 0.3
	**At inoculation**	0.05	26	39	25	66
		0.5	26	24	25	70
		1.0	76	74	75	81
		2.0	86	85	85	90
	**LC**_**50**_	-	0.79 (0.69–0.89)	0.83 (0.72–0.98)	0.70 (0.58 − 0.82)	0.61 (0.49–0.71)
	**Slop ± SE**	-	2.9 ± 0.2	2.9 ± 0.3	2.4 ± 0.3	2.4 ± 0.3
	**A week after inoculation**	0.05	18	12	15	53
		0.5	29	29	26	51
		1.0	25	28	10	83
		2.0	49	52	49	100
	**LC**_**50**_	-	2.1 (1.6 − 3.6)	1.9 (1.5–2.9)	2.2 (1.7–3.5)	0.5 (0.35–0.62)
	**Slop ± SE**	-	1.5 ± 0.3	1.8 ± 0.3	1.7 ± 0.3	2.4 ± 0.3
**Control**		-	-	-	-	-

LC_50_ was calculated according to Proban software program.

### Effect of different DE concentrations on plant growth of eggplant infected with root knot nematode

**Fig. 2:**
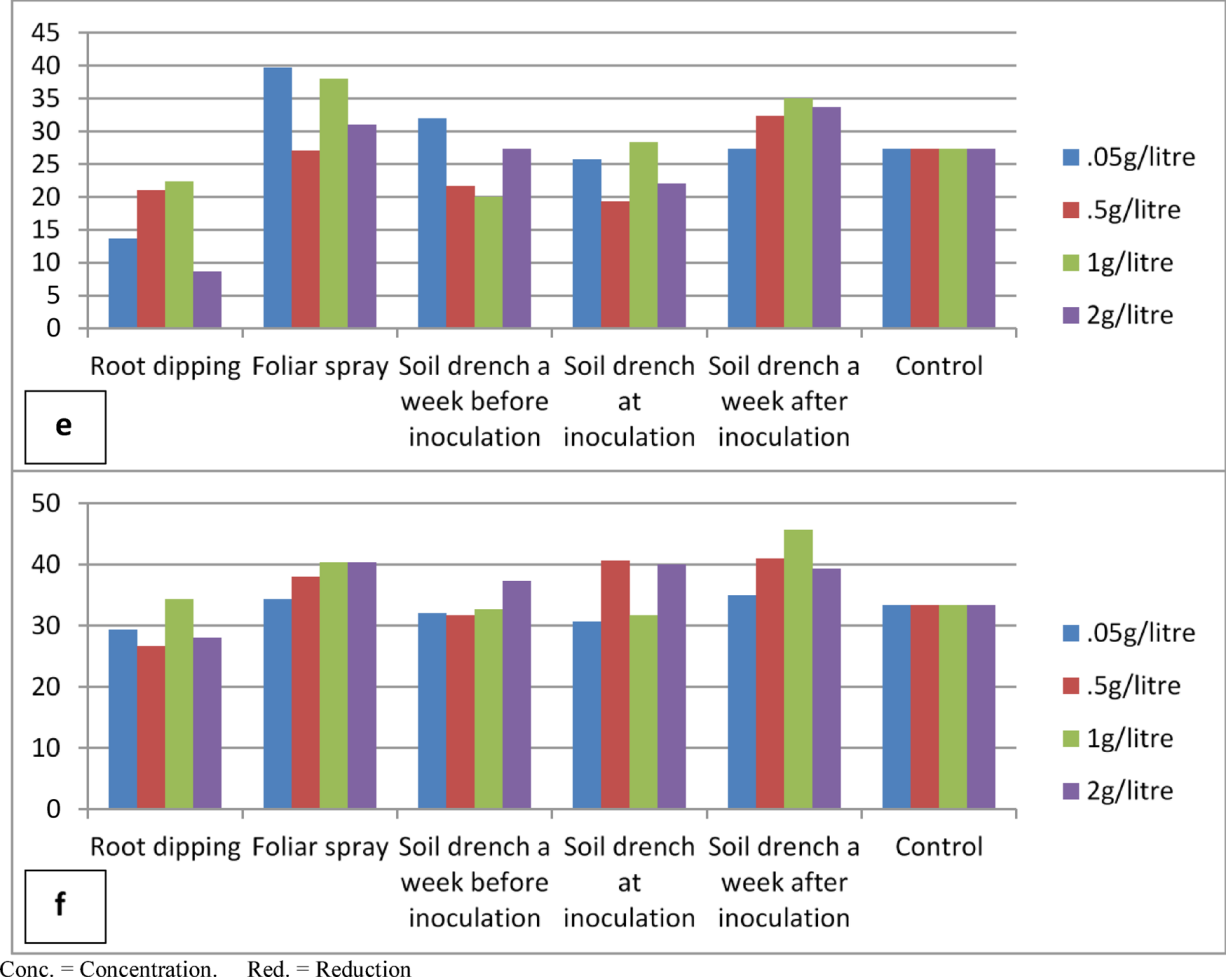
Effect of different DE concentrations (0.05,0.5,1.0 and2.0 g/L) on root-lengths **(e)** and shoot-lengths **(f)** of eggplant infected with root knot nematode, *Meloidogyne javanica*.

**Fig. 3:**
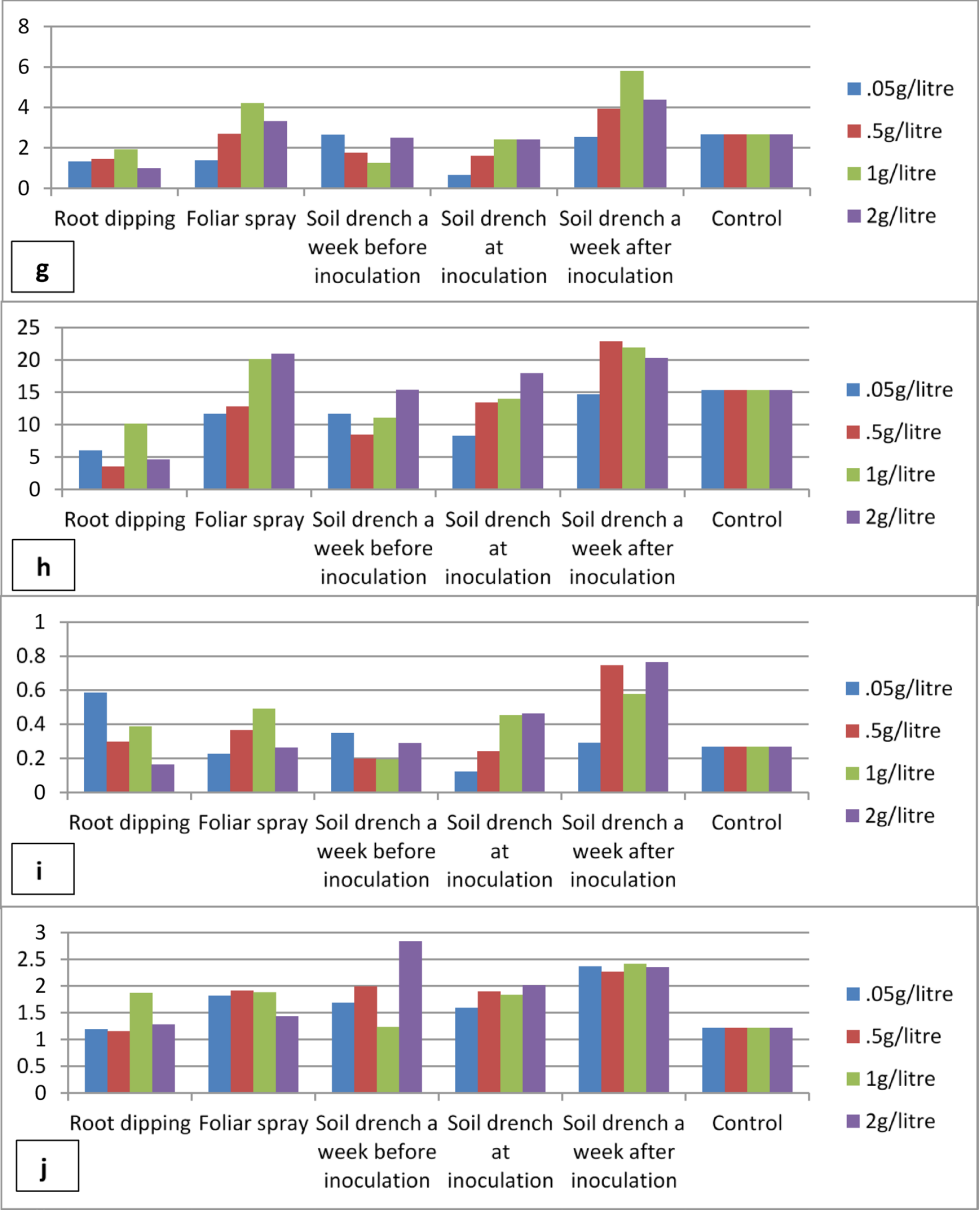
Effect of different DE concentrations (0.05,0.5,1.0 and2.0 g/L) on root-fresh weights **(g)**, shoot-fresh weights **(h)**, root-dry weights **(i)** and on shoot-dry weights **(j)** of eggplant infected with root knot nematode, *Meloidogyne javanica.*

The use of DE in controlling root knot nematode, *M. javanica* led to improvement in he growth parameters of eggplant which were increased particularly as root and shoot dry weights as shown in (Table 4; Figs. 2 and 3). Among application methods, soil drench applied one week after nematode inoculation resulted in the highest percentages of increase across most growth parameters compared to other methods and the untreated control. Root dipping produced the lowest increase percentages in all growth parameters among the tested methods.

Foliar spraying at 0.05 and 1.0 g/L showed root length increases of 45 and 39%, respectively, while one week after inoculation method recorded the highest increase percent by 37% in shoot length at 1.0 g/L and improved root fresh weight by 118% and 64% at 1.0 and 2.0 g/L, respectively, when foliar application at the same concentrations achieved 58% and 25%. As for shoot fresh weight, soil treatment one week after inoculation at 0.5 and 1.0 g/L recorded increase percentages by 50% and 43%, followed by a 37% observed with foliar spray at 2.0 g/L.

As for root dry weight soil treatment applied one week after inoculation produced increases of 178%, 115%, and 185% at 0.5, 1.0, and 2.0 g/L, respectively relative to the control, when foliar spray resulted in an 83% increase at at 1.0 g/L. The highest increase percent of 133% was observed with soil treatment applied one week before inoculation at 2.0 g/L followed by soil treatment applied one week after inoculation which increased shoot dry weight across all DE concentration by 94%, 86%, 99%, and 93%, respectively.

**Table 4:** Effect of varying diatomaceous earth (DE) concentrations on the growth of eggplant (*Solanum melongena* L.) infected with the root-knot nematode *Meloidogyne javanica*.

Types of application	Conc. g/L	Lengths (cm)				Fresh weights (g)				Dry weights (g)			
		Root	%Inc.	Shoot	% Inc.	Root	% Inc.	Shoot	% Inc.	Root	% Inc.	Shoot	% Inc.
**Root dipping**	0.05	13.67 gh	-	29.33 cd	-	1.33 de	-	6.02 ef	-	0.250 a	-	1.089 b	-
	0.5	21.00 ef	-	26.67 e	-	1.45 de	-	3.52 f	-	0.297 a	11	1.156 b	-
	1.0	22.33 de	-	34.33 bc	3	1.93 bc	-	10.14 cd	-	0.387 a	44	1.867 ab	53
	2.0	8.67 h	-	28.00 de	-	0.99 ef	-	4.62 ef	-	0.497 a	85	1.282 ab	5
**Foliar spray**	0.05	39.67 a	45	34.33 bc	3	1.38 de	-	11.66 ab	-	0.227 a	-	1.819 ab	49
	0.5	27.00 cd	-	38.00 ab	14	2.69 bc	1	12.78 ab	-	0.365 a	36	1.910 ab	57
	1.0	38.00 ab	39	40.33 ab	21	4.20 ab	58	20.15 ab	32	0.491 a	83	1.881 ab	54
	2.0	31.00 ab	13	40.33 ab	21	3.32 bc	25	20.95 ab	37	0.265 a	-	1.431 ab	17
**Application a week before inoculation**	0.05	31.67 ab	16	32.00 bc	-	2.65 bc	-	11.64 ab	-	0.348 a	30	1.864 ab	53
	0.5	21.67 de	-	31.67 bc	-	1.76 cd	-	8.44 de	-	0.199 a	-	1.989 ab	63
	1.0	20.00 fg	-	32.67 bc	-	1.26 ef	-	11.06 bc	-	0.193 a	-	1.235 ab	1
	2.0	27.33 cd	-	37.33 ab	12	2.49 bc	-	15.37 ab	0.4	0.289 a	8	2.835 a	133
**Application at inoculation**	0.05	25.67 cd	-	30.67 bc	-	0.66 f	-	8.25 de	-	0.456 a	70	1.588 ab	30
	0.5	19.33 fg	-	40.67 ab	22	1.60 de	-	13.38 ab	-	0.242 a	-	1.893 ab	55
	1.0	28.33 bc	4	31.67 bc	-	2.41 bc	-	13.96 ab	-	0.452 a	69	1.833 ab	50
	2.0	22.00 de	-	40.00 ab	20	2.41 bc	-	17.96 ab	17	0.463 a	73	2.013 ab	65
**Application a week after inoculation**	0.05	27.33 cd	-	35.00 ab	5	2.54 bc	-	14.67 ab	-	0.291 a	9	2.364 ab	94
	0.5	32.33 ab	18	41.00 ab	23	3.92 ab	47	22.89 a	50	0.746 a	178	2.264 ab	86
	1.0	35.00 ab	28	45.67 a	37	5.80 a	118	21.91 ab	43	0.577 a	115	2.410 ab	99
	2.0	33.67 ab	23	39.33 ab	18	4.37 ab	64	20.32 ab	33	0.765 a	185	2.348 ab	93
**Control**	-	27.33 cd	-	33.33 bc	-	2.66 bc	-	15.31 ab	-	0.268 a	-	1.218 ab	-
**F value**	-	5.7397	-	2.3373	-	0.00000	-	2.9597	-	0.9003	-	0.954	
**P value**	-	0.15730	-	0.06556	-	2.8554	-	0.01664	-	0.00000	-	0.00000	-

Each value is the mean of four replicates. Means within a column followed by the same letter(s) are not significantly different according to Duncan’s Multiple Range Test (*p* ≤ 0.05). Abbreviations: Conc. = Concentration; Inc. = Increase.

## Discussion

Diatomaceous Earth (DE), owing to its high SiO_2_ content, has emerged as a novel strategy for managing insect pests in field crops^30,31^. Due to the limited research on the effects of diatomaceous earth on plant-parasitic nematodes, the present discussion draws on evidence from studies on insects and stored-grain pests to explain the potential mechanism of DE activity against root-knot nematodes.

DE is widely regarded as one of the most effective natural dust insecticides. When insects traverse treated surfaces, DE particles become trapped on their bodies. The generally accepted mode of action is a desiccating effect on insects^32,33^, indicating that toxicity is primarily physical rather than chemical in nature^34^. Several studies have concluded that the primary mechanism involves the absorption of epicuticular lipids and fatty acids, leading to desiccation in arthropods^20,35,36^.

Based on this mechanism observed in insect larvae, it can be hypothesized that DE acts present study, in which direct exposure of nematode juveniles to different DE concentrations for 24, 48, and 72 h in laboratory tests resulted in larval dehydration and deformation. The adhesion of DE granules to juvenile bodies and subsequent absorption of water and fatty acids likely contributed to the observed 100% mortality rates at most concentrations. These findings are consistent with those of Öztekin and Mutlu^37^, who evaluated three DE formulations two local (Aydın and Ankara) and one commercial (Silicosec^®^; Biofa, Germany) against *Callosobruchus maculatus* (F.) (Coleoptera: Bruchidae) adults on beans at doses of 200, 400, and 600 mg/kg over 2, 3, 5, 7, 14, and 21 days. They reported that Ankara DE achieved the highest mortality rate (100%) after 5 days at 25 °C with 600 ppm, followed by Aydın (84.97%) and SilicoSec^®^ (63.47%).

The same mechanism may explain the results of the pot experiment. Root dipping for one hour and soil drench at the time of inoculation, across all tested DE concentrations (0.05, 0.5, 1.0, and 2.0 g/L), produced the highest reductions in the number of root galls, females, and egg masses compared to other application methods. Soaking the roots likely allowed DE granules to be absorbed, adversely affecting juveniles and impairing their ability to penetrate roots and establish infection. Similarly, soil drench applied concurrently with nematode inoculation resulted in high reduction rates for the same parameters, particularly at the higher concentrations (1.0 and 2.0 g/L). Foliar spraying and the two soil treatments applied before and after inoculation produced moderate reduction rates.

The observed improvements in most growth parameters may also be attributed to DE acting as a beneficial soil amendment. By improving soil aeration and water retention, DE can promote stronger root development and enhance the habitat for nematode-suppressing microbes^23^.

These findings are in agreement with previous studies. For example, Mahmoud et al.^38^ reported that a combination of 10 mL/L Neem Azal-T/S (commercial neem) and 2.0 g/L DE applied as a foliar spray significantly reduced *Myzus persicae* (Sulzer) infestations on artichoke, ranking first in alleviating aphid damage and improving vegetative growth and bud yield, followed by neem alone, while DE alone was the least effective. This aligns with our results, which demonstrated the role of DE in improving eggplant growth, particularly root and shoot dry weight.

Consistent with prior findings, the combination of Neem Azal-T/S and DE also achieved superior control of *Aphis craccivora* on yardlong beans^39^. Furthermore, DE particularly when co-applied with *Paecilomyces lilacinus* at 2.3 × 10⁹ conidia/mL provided enhanced suppression of *Thrips tabaci* Lind. on cotton under both laboratory and semi-natural conditions^40^. Building on this foundation, the present study targeting the root-knot nematode *Meloidogyne javanica* yields results that are consistent with these earlier observations.

## Conclusion

This study provides the first comprehensive evidence on the efficacy of diatomaceous earth (DE) against *Meloidogyne javanica* in eggplant under greenhouse conditions. DE achieved 100% mortality of second-stage juveniles at a concentration of 0.05 g/L and 85% mortality at 0.01 g/L in laboratory tests. Root dipping at 2.0 g/L resulted in reductions exceeding 90% in gall formation, egg masses, and adult females. Soil drench applied at the time of inoculation also suppressed nematode parameters and was associated with increases in shoot dry weight. The results indicate that DE offers a physical mode of action against nematodes, as opposed to the chemical toxicity of conventional nematicides. The application methods tested particularly root dipping and soil drench demonstrated varying levels of efficacy, with root dipping producing the highest reductions in nematode parameters. The observed improvements in plant growth parameters suggest that DE may also function as a soil amendment, although further investigation is required to confirm this effect. Several limitations should be acknowledged. Field validation is necessary, as factors such as soil texture, moisture, organic matter content, and microbial communities may influence DE performance; its abrasive activity is known to diminish under wet conditions. Additionally, these findings are specific to *M. javanica* and eggplant under greenhouse conditions; efficacy may differ for other *Meloidogyne* species, crops, and production systems.

Future research should include field-scale evaluations across diverse agroecological conditions, long-term soil health monitoring, and investigations into potential synergistic effects when DE is combined with other physical control agents. Within the boundaries of this study, DE represents a potential component for integrated root-knot nematode management that warrants further investigation.

## Data Availability

All data supporting the findings of this study are available within the paper.

## References

[1] Mahanta, C. L. & Kalita, D. Eggplant. In Nutritional Composition and Antioxidant Properties of Fruits and Vegetables (ed Jaiswal, A. K.) 273–287 (Academic, (2020).

[2] Chen, X. et al. Comparative transcriptome analysis provides insights into molecular mechanisms for parthenocarpic fruit development in eggplant (*Solanum melongena* L). *PLOS One*. **12**, e0179491 (2017). 28604820 10.1371/journal.pone.0179491 PMC5467848

[3] El-Qurashi, M. A., Al-Yahya, F., Almasrahi, A. & Shakeel A.Growth and resistance response of eleven eggplant cultivars to infection by the Javanese root-knot nematode – *Meloidogyne javanica* under greenhouse conditions. *Plant Prot. Sci.* **61** (3), 291–300 (2025).

[4] Noling, J. W. Nematode Management in Tomatoes, Peppers and Eggplant. ENY-032/NG032 (University of Florida, Institute of Food and Agricultural Sciences, Florida Cooperative Extension Service, (2002).

[5] Shaaban, M. H., Mahdy, M. E., Selim, M. E., Mousa, E. M. & Pathological Chemical and Molecular Analysis of Eggplant Varieties Infected with Root-knot Nematodes (Meloidogyne spp). *Egypt. J. Crop Prot.* **18** (1), 1–13 (2023).

[6] Yadav, H., Roberts, P. A. & Lopez-Arredondo, D. Combating Root-Knot Nematodes (*Meloidogyne* spp.): From Molecular Mechanisms to Resistant Crops. *Plants* **14** (9), 1321 (2025). 40364350 10.3390/plants14091321 PMC12073475

[7] Khan, A., Ahmad, G., Haris, M. & Khan, A. A. Bio-organics management: novel strategies to manage root-knot nematode, *Meloidogyne incognita* pest of vegetable crops. *Gesunde Pflanzen*. **75**, 193–209 (2023b).

[8] Chariot, P. L. & Steinmetz, N. F. Delivery of pesticides to plant parasitic nematodes using tobacco mild green mosaic virus as a nanocarrier. *ACS Nano*. **11**, 4719–4730 (2017). 28345874 10.1021/acsnano.7b00823

[9] Kenney, E. & Eleftherianos, I. Entomopathogenic and plant pathogenic nematodes as opposing forces in agriculture. *Int. J. Parasitol.* **46**, 13–19 (2016). 26527129 10.1016/j.ijpara.2015.09.005 PMC4707073

[10] Mehak, A. et al. Titanium dioxide nanoparticles as a sustainable strategy to control root-knot nematodes (RKNs) in tomato (*Solanum lycopersicum* L). *Physiol. Mol. Plant Pathol.* **143**, 103122 (2026).

[11] Afzal, A. & Mukhtar, T. Revolutionizing nematode management to achieve global food security goals - An overview. *Heliyon* **10**, e25325 (2024). 38356601 10.1016/j.heliyon.2024.e25325 PMC10865254

[12] Saeed, M., Mukhtar, T., Ahmed, R., Mehmood, U. & Iqbal, M. A. Synergistic efficacy of *Azadirachta indica* and biocontrol agents in mitigating *Meloidogyne javanica* infection in peach. *Australas. Plant Pathol.* **55**, 14 (2026).

[13] Azeem, W. et al. The nematicidal potential of *Moringa oleifera* extracts and rhizobacteria against Meloidogyne incognita in tomato. *Front. Plant Sci.* **16**, 1562074 (2025). 40433158 10.3389/fpls.2025.1562074 PMC12106468

[14] Tariq-Khan, M. et al. Population Dynamics and Virulence Patterns of Root-Knot Nematodes (*Meloidogyne* spp.) on Tomato in Poonch Highlands, Azad Jammu and Kashmir, Pakistan. *J. Phytopathol.* **173** (2), e70060 (2025).

[15] Meel, S. & Saharan, B. S. Microbial warfare against nematodes: A review of nematicidal compounds for horticulture, environment, and biotechnology. *Microbe* **9**, 100557 (2025).

[16] Khan, M. et al. Counter-attack of biocontrol agents: Environmentally benign Approaches against Root-knot nematodes (*Meloidogyne* spp.) on Agricultural crops. *Heliyon* **9**, e21653 (2023a). 37954375 10.1016/j.heliyon.2023.e21653 PMC10632526

[17] Nikpay, A. Diatomaceous earths as alternatives to chemical insecticides in stored grain. *Insect Sci.* **13** (6), 421–429 (2006).

[18] Abd El-Latef, E. A., Wahba, M. N., Mousa, S. M., El-Bassyouni, G. T. & El-Shamy, A. M. Cu-doped ZnO-nanoparticles as a novel eco-friendly insecticide for controlling Spodoptera littoralis. *Biocatal. Agric. Biotechnol.* **52**, 102823 (2023).

[19] Wahba, T. F., Wahba, M. N., Abo-Elfadl, M. T., El-Bassyouni, G. T. & Mousa, S. M. Efficacy and characterization of synthesized metal oxide nanoparticles and diatomaceous earth on stored product insect control. *J. Stored Prod. Res.* **112**, 102622 (2025).

[20] Shah, M. A. & Khan, A. A. Use of Diatomaceous Earth for the Management of Stored Product Pests. *Int. J. Pest Manage.* **60** (02), 1–14 (2014).

[21] De Tommasi, E., Gielis, J. & Rogato, A. Diatom Frustule Morphogenesis and Function: A Multidisciplinary. *Surv. Mar. Genomics*. **35** (1), 1–18 (2017). 10.1016/j.margen.2017.07.001 28734733

[22] Khan, A. et al. Biocontrol of root knot nematodes by endophytic fungus isolated from garlic. *Sci. Hort.* **332**, 113223 (2024).

[23] Makeleni, S., Manyevere, A., Mashamaite, C. V. & Ramatsitsi, N. Soil borne nematodes as bioindicators of soil health in cropping systems: a systematic review. *Arch. Agron. Soil. Sci.* **71** (1), 1–21 (2025).

[24] Cucu, M. A., Choudhary, R., Trkulja, V., Garg, S. & Matić, S. Utilizing Environmentally Friendly Techniques for the Sustainable Control of Plant Pathogens: A Review. *Agronomy* **15** (7), 1551 (2025).

[25] Taylor, A. L., Propkin, V. H. & Martin, G. O. Perineal patterns of root-knot nematodes. *Phytopathology* **45**, 26–34 (1955).

[26] Abbott, W. S. A method of computing the effectiveness of an insecticide. *J. Econ. Entomol.* **18**, 265–267 (1925). 3333059

[27] Cobb, N. A. Estimating the nematode population of the soil. *Agricultural Technol. circular.* **48,** 1–48 (1918).

[28] Christie, J. R. & Perry, V. G. Removing nematodes from soil. *Proc. Helminthological Soc. Wash.* **18**, 106–108 (1951).

[29] De Silva, F. A. S. & de Azevedo, C. A. V. The Assistat Software Version 7.7 and its use in the analysis of experimental data. *Afr. J. Agric. Res.* **11** (39), 3733–3740 (2016).

[30] Athanassiou, C. G., Arthur, F. H., Opit, G. P. & Throne, J. E. Insecticidal Effect of Diatomaceous Earth Against Three Species of Stored-Product Psocids on Maize, Rice, and Wheat. *J. Econ. Entomol.* **102** (4), 1673–1680 (2009). 19736783 10.1603/029.102.0435

[31] Wakil, W., Ashfaq, M., Ghazanfar, M. U. & Riasat, T. Susceptibility of stored-product insects to enhanced diatomaceous earth. *J. Stored Prod. Res.* **46**, 248–249 (2010).

[32] Ebeling, W. Sorptive dust for pest control. *Ann. Rev. Entomol.* **16**, 123–158 (1971). 4324088 10.1146/annurev.en.16.010171.001011

[33] Mewis, I. & Reichmuth, C. Diatomaceous earths against the Coleoptera: granary weevil Sitophilus granarius (Curculionidae), the confused flour beetle Tribolium confusum (Tenebrionidae), and the mealworm Tenebrio molitor (Tenebrionidae). In Proceedings of the 7th International Conference on Stored-Product Protection (eds. Zuxun, J., Quan, L., Yongsheng, L., Xianchang, T. & Lianghua, G.) 765–780Sichuan Publishing House of Science and Technology, Chengdu, China, (1998).

[34] Subramanyam, B. & Roesli, R. Inert dusts. In Alternatives to Pesticides in (eds Stored-Product, I. P. M., Subramanyam, B. & Hagstrum, D. W.) 321–373 (Kluwer Academic, Dordrecht, (2000).

[35] Mewis, I. Ulrichs,Ch. Wirkungsweise amorpher Diatomeenerden auf vorratsschädliche Insekten. Untersuchung der abrasiven und sorptiven Effekte. *J. Pest Sci.* **72**, 113–121 (1999).

[36] Prasantha, B. D. R. & Toxicological biological and physiological effects of diatomaceous earths on the bean weevil Acanthoscelides obtectus (Say) and the cowpea weevil Callosobruchus maculatus (F.) (Coleoptera: Bruchidae). Ph.D. thesis, Humboldt University of Berlin, Berlin, Germany (2003).

[37] Öztekin, N. & Mutlu, Ç. Efficacy of three diatomaceous earth formulations against cowpea beetle, Callosobruchus maculatus (F.) (Coleoptera: Bruchidae), on bean. *J. Entomol. Zool. Stud.* **8** (6), 147–152 (2020).

[38] El-Wakeil, N. E. & Saleh, S. A. Effects of neem and diatomaceous earth against *Myzus persicae* and associated predators in addition to indirect effects on artichoke growth and yield parameters. *Archives Phytopathol. Plant. Prot.* **42**, 1132–1143 (2009).

[39] Ulrichs, C. H., Mewis, I. & Schitzler, W. H. Efficacy of neem and diatomaceous earth against cowpea aphids and their deleterious effect on Coccielidae. *J. Appl. Entomol.* **125** (9–10), 571–575 (2001).

[40] Wakil, W., Ghazanfar, M. U., Kwon, Y. J., Islam, S. U. & Ali, K. Toxicity of *Paecilomyces lilacinus* blended with non-conventional agents to control cotton thrips (Thrips tabaci Lind.) (Insecta: Thysanoptera: Thripidae). *Afr. J. Microbiol. Res.* **6** (3), 526–533 (2012).

